# Drug-induced herpes zoster: a pharmacovigilance analysis of FDA adverse event reports from 2004 to 2024

**DOI:** 10.3389/fphar.2025.1565480

**Published:** 2025-03-26

**Authors:** Jiali Xia, Jing Zhang, Hongyu Zhu, Li Ding

**Affiliations:** ^1^ Department of Dermatology, The Affiliated Xuzhou Municipal Hospital of Xuzhou Medical University, Xuzhou, China; ^2^ Department of Critical Care Medicine, The Affiliated Xuzhou Municipal Hospital of Xuzhou Medical University, Xuzhou, China; ^3^ Central Laboratory, The Affiliated Xuzhou Municipal Hospital of Xuzhou Medical University, Xuzhou, China

**Keywords:** herpes zoster, adverse events, FAERS, real-world data analysis, pharmacovigilance

## Abstract

**Background:**

Herpes zoster severely impacts patients’ quality of life and therapeutic results. This research utilized data from the FDA Adverse Event Reporting System (FAERS) to examine the prevalence and attributes of drug-induced herpes zoster.

**Methods:**

We analyzed FAERS reports about zoster from Q1 2004 to Q3 2024 and developed a list of possible pathogenic agents. Ranked the 30 medicines with the greatest incidence of reported herpes zoster cases. Statistical disproportionality analysis was employed to identify an elevated reporting frequency of herpes zoster linked to a particular medication.

**Results:**

Herpes zoster was referenced in 50,164 FAERS reports from 2004 to 2024. The majority of the implicated drugs were immunosuppressants. Anifrolumab exhibited the greatest ROR and PRR ratings among the drugs evaluated. Furthermore, rozanolixizumab, tozinameran, elapegademase, and other medications not indicated for inducing herpes zoster were recognized, underscoring the necessity for increased clinical vigilance and awareness. Nonetheless, these correlations should be regarded with caution, as they do not establish a direct causative relationship.

**Conclusion:**

This study underscores the need of pharmacovigilance in recognizing and comprehending drug-induced herpes zoster. Additional research is required to validate these findings and to design strategies for risk management and reduction to enhance treatment outcomes in patients.

## 1 Introduction

Herpes zoster (HZ) is an infectious disease resulting from the reactivation of the dormant varicella-zoster virus (VZV). Severe neuralgia and a unilaterally dispersed vesicular rash along dermatomes are the hallmarks of HZ. The occurrence of HZ is generally associated with a decline in host immune function, with advanced age, immunosuppressive diseases, and certain medications being major risk factors.

According to epidemiological research, the global annual incidence of HZ ranges from 3 to 5 cases per 1,000 individuals (Kawai et al., 2014). The incidence, which can reach 10 to 12 instances per 1,000 people, is much higher among the elderly and immunocompromised populations (Tseng et al., 2020). With an annual increase of 2.5%–5.0% (Kawai et al., 2016), the incidence of HZ in the Asia-Pacific region ranges from 3 to 10 cases per 1,000 person-years (Chen et al., 2017). The hospitalization rate for HZ varies between 2 and 25 per 100,000 person-years worldwide, whereas the mortality rate varies between 0.017 and 0.465 per 100,000 person-years, and the recurrence rate is between 1% and 10% (Kawai et al., 2014; Tseng et al., 2020). This disease not only increases the health burden on affected individuals but also places substantial strain on healthcare system resources.

The impact of HZ extends far beyond its acute symptomatic phase. According to a meta-analysis, the yearly incidence of postherpetic neuralgia (PHN), a chronic pain illness that significantly reduces quality of life, varies between 3.9 and 42.0 cases per 100,000 people (van Hecke et al., 2014). Patients with HZ commonly experience decreased work productivity, increased psychological stress, and impaired social functioning, with these effects potentially persisting for months or even years.

Immunosuppression is a recognized risk factor for herpes zoster, and emerging evidence indicates that some medications may elevate the risk of herpes zoster through several mechanisms, including immunological modulation or direct effects on viral reactivation (Tran et al., 2017; Chakravarty, 2017). Immunosuppressants (Murdaca et al., 2019; Hu et al., 2016), corticosteroids (Qian et al., 2021), and anticancer drugs (Serra et al., 2022) have been consistently linked to an elevated incidence of HZ. These medications may suppress the host’s cellular immune response, reducing the body’s ability to control VZV and thus increasing the likelihood of viral reactivation. In recent years, biologic agents such as anti-TNF-α drugs (Steiger et al., 2022) and JAK inhibitors (Jeong et al., 2022) have also been reported to be associated with an increased risk of HZ. Studies have found that the risk of HZ is significantly elevated in patients with rheumatoid arthritis treated with TNF-α or JAK inhibitors, with risk ratios of 1.63 and 3.66, respectively (Redeker et al., 2022).

However, research on drug-induced HZ remains insufficient, particularly systematic analyses based on large-scale real-world data. Although a small number of studies have focused on assessing the risk of HZ associated with specific drugs, these studies are mostly limited to single agents and have not yet formed a comprehensive risk assessment of multiple medications. Moreover, the causal relationship between drugs and the risk of HZ lacks support from large-sample, multicenter data.

Post-marketing surveillance is a method for identifying associations between drugs and adverse reactions. Since 2004, a significant number of adverse event reports have been gathered by the voluntary FAERS adverse event database, which is a useful tool for mining medication risk signals (Raschi et al., 2019). Prior research has effectively employed FAERS data to detect drug safety signals, including cystitis (Zheng et al., 2024), sarcopenia (Zhang and Yao, 2024), and hypoglycemia (Li et al., 2024).

Still, there is a lack of systematic research on the risk of drug-induced HZ using FAERS data. Thus, this work intends to fill this research void by utilizing FAERS data and using techniques for signal detection to find high-risk medications linked to HZ. The results will support pharmacovigilance and risk management tactics by giving physicians and drug regulatory bodies scientific proof. The study may ultimately lead to better patient outcomes and a decrease in the burden of drug-induced HZ cases.

## 2 Methods

### 2.1 Data collection

This retrospective pharmacovigilance study was utilized data from the FAERS. The FAERS database is composed of seven primary datasets: demographic information of patients (DEMO), drug and biological product information (DRUG), adverse events (REAC), patient outcomes (OUTC), report sources (RPSR), start and end dates of drug therapy (THER), and indications for drug use (INDI). We extracted the American Standard Code for Information Interchange (ASCII) reporting files from the FAERS database, covering the first quarter of 2004 through the third quarter of 2024.

### 2.2 Data extraction and analysis

In FAERS, adverse event (AE) information is standardized using the Medical Dictionary for Regulatory Activities (MedDRA) preferred terms (PTs). We searched for “herpes zoster” in the Preferred Term (PT) field to identify drugs that caused herpes zoster and downloaded all relevant reports. The generic names of drugs were used as the unique identifiers for statistical evaluation. Relevant clinical characteristics such as sex, age, reporter, reporting region, patient outcomes, and indications were collected. Our institution deemed that ethical approval was not necessary, as individual patient identification was not feasible.

Individual safety reports (ISRs) were counted, with one ISR equal to one AE report, and the 30 most frequently reported drugs associated with herpes zoster were screened. Proportional reporting ratio (PRR) and reporting odds ratio (ROR) analyses were used to postulate potential associations between drugs and herpes zoster. PRR estimates relative risk but may be sensitive yet prone to false positives, especially when the number of reported cases is small. Conversely, ROR provides more consistent estimates of risk ratios with less bias. Higher ROR and PRR values indicate stronger associations between drugs and related adverse events. This study combined both ROR and PRR algorithms, leveraging their respective strengths to enhance detection and validate results from different perspectives. The combined use of these algorithms allows for cross-validation, reduces false positives, and improves the detection of potentially rare adverse reactions through threshold and variance adjustments. The specific formulas and thresholds for all algorithms are shown in Table 1. Ultimately, effective ADR results should meet the positive signal selection criteria outlined by both algorithms mentioned above. The information was thoroughly processed and inspected using applications such as Excel and R Studio.

**TABLE 1:** Summary of algorithms used for signal detection.

Algorithms	Formula	Threshold
ROR	ROR = ad/b/c	lower limit of 95% CI > 1, N ≥ 3
	95%CI = eln (ROR)±1.96 (1/a+1/b+1/c+1/d)^0.5	
PRR	PRR = a (c + d)/c/(a+b)	PRR≥2, χ2≥4, N ≥ 3
	χ2 = [(ad-bc)^2](a+b + c + d)/[(a+b) (c + d) (a+c) (b + d)]	

^a^ number of reports containing both the suspect drug and the suspect adverse event.

^b^ number of reports containing the suspect adverse event with other medications (except the drug of interest).

^c^ number of reports containing the suspect drug with other adverse drug reactions (except the event of interest).

^d^ number of reports containing other drugs and other adverse events. ROR, reporting odds ratio; PRR, proportional reporting ratio; CI, confidence interval; χ 2, chi-squared.

## 3 Results

### 3.1 Descriptive analysis

**FIGURE 1:**
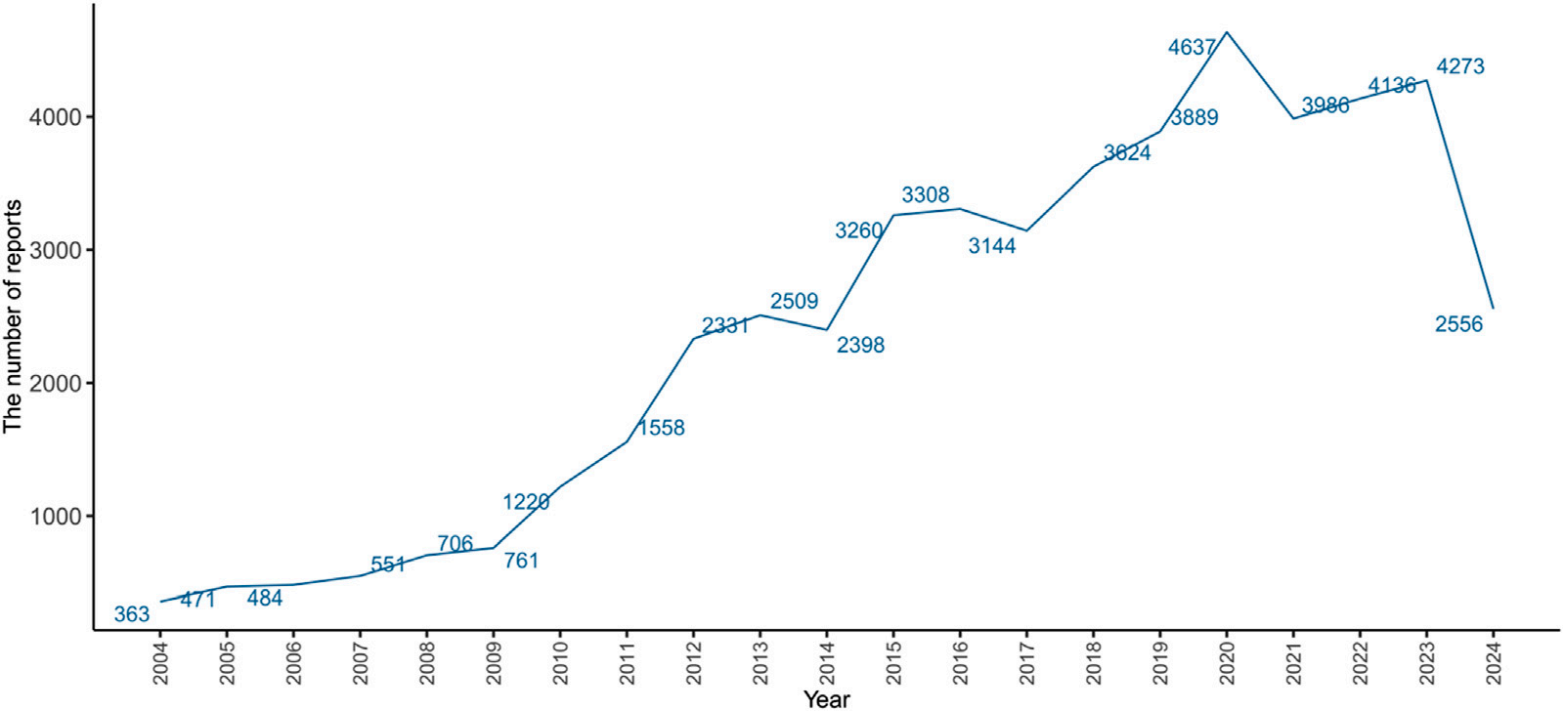
Number of reported cases of drug-induced herpes zoster from Q1 2004 to Q3 2024. Data for 2024 is incomplete, representing reports only up to the third quarter (Q3).

From the first quarter of 2004 to the third quarter of 2024, the FAERS database recorded AE reports, of which 50,164 cases were related to herpes zoster. As shown in Figure 1, the epidemiological trend of reported herpes zoster cases has been on the rise since 2004, reaching a peak in 2020 with 4,637 cases. It is important to note that the data for 2024 only includes reports up to the third quarter (Q3), which likely accounts for the lower number of reported cases compared to previous full years. Table 2 lists the clinical characteristics of these 50,1646 reports. Herpes zoster was more likely to occur in patients aged 41–65 years (31.11%), followed by patients over 65 years (26.05%), 19–41 years (7.46%), and 18 years or younger (1.33%). The incidence was higher in females (64.41%) than in males (26.12%). The most frequently reported outcomes were hospitalization (34.05%), followed by death (5.02%), disability (2.79%), life-threatening conditions (2.21%), requiring intervention to prevent permanent impairment (0.25%), and congenital anomalies (0.03%). Most reports were submitted by consumers (21679 reports, 43.22%), with the United States being the primary reporting country (25420 reports, 50.67%). Multiple sclerosis (4,124 reports, 7.24%) and rheumatoid arthritis (8,802 reports, 15.45%) were the most common indications.

**TABLE 2:** Clinical characteristics of reported drug-induced herpes zoster.

Variable	Reports, n (%)
Sex	
female	32309 (64.41)
male	13104 (26.12)
unknown	4751 (9.47)
Age	
<19	669 (1.33)
19–41	3744 (7.46)
41–65	15608 (31.11)
≥65	13068 (26.05)
unknow	17075 (34.04)
Reporter	
Consumer	21679 (43.22)
Physician	13012 (25.94)
Pharmacist	7337 (14.63)
Other health-professional	6084 (12.13)
unknown	1891 (3.77)
Lawyer	118 (0.24)
Registered Nurse	43 (0.09)
Reported countries (Grouped by Continent)	
North America	
United States	25420 (50.67)
Canada	5597 (11.16)
Puerto Rico	55 (0.11)
Mexico	52 (0.10)
Europe	
Germany	1419 (2.83)
United Kingdom	1019 (2.03)
France	518 (1.03)
Italy	347 (0.69)
Spain	245 (0.49)
Netherlands	141 (0.28)
Poland	126 (0.25)
Czechia	107 (0.21)
Belgium	100 (0.20)
Austria	99 (0.20)
Portugal	96 (0.19)
Israel	90 (0.18)
Switzerland	85 (0.17)
Greece	81 (0.16)
Ireland	78 (0.16)
Sweden	74 (0.15)
Asia	
Japan	1948 (3.88)
China	325 (0.65)
Korea, South	150 (0.30)
Turkey	70 (0.14)
India	53 (0.11)
South America	
Brazil	422 (0.84)
Colombia	182 (0.36)
Argentina	116 (0.23)
Oceania	
Australia	362 (0.72)
Other/Unknown	
Unspecified Country	107 (0.21)
other	10680 (21.29)
Outcomes	
other serious	23201 (55.64)
hospitalization	14199 (34.05)
death	2092 (5.02)
disability	1162 (2.79)
life threatening	922 (2.21)
required intervention to Prevent Permanent Impairment/Damage	106 (0.25)
congenital anomaly	13 (0.03)
Indications	
acute lymphocytic leukaemia	113 (0.20)
acute myeloid leukaemia	59 (0.10)
ankylosing spondylitis	528 (0.93)
anxiety	114 (0.20)
arthralgia	74 (0.13)
arthritis	177 (0.31)
asthma	777 (1.36)
atrial fibrillation	93 (0.16)
b-cell lymphoma	124 (0.22)
back pain	102 (0.18)
blood cholesterol increased	203 (0.36)
blood pressure	61 (0.11)
bone marrow conditioning regimen	69 (0.12)
breast cancer	292 (0.51)
breast cancer female	98 (0.17)
breast cancer metastatic	158 (0.28)
cardiac failure	52 (0.09)
chronic hepatitis c	69 (0.12)
chronic lymphocytic leukaemia	452 (0.79)
chronic myeloid leukaemia	154 (0.27)
chronic obstructive pulmonary disease	245 (0.43)
colitis ulcerative	870 (1.53)
colon cancer	50 (0.09)
complex regional pain syndrome	69 (0.12)
constipation	52 (0.09)
crohn’s disease	2051 (3.60)
depression	217 (0.38)
dermatitis atopic	477 (0.84)
dermatomyositis	61 (0.11)
diabetes mellitus	245 (0.43)
diffuse large b-cell lymphoma	136 (0.24)
drug use for unknown indication	342 (0.60)
fibromyalgia	109 (0.19)
follicular lymphoma	53 (0.09)
gait disturbance	51 (0.09)
gastrooesophageal reflux disease	153 (0.27)
haemolytic uraemic syndrome	50 (0.09)
hepatitis c	141 (0.25)
herpes zoster	256 (0.45)
hidradenitis	68 (0.12)
hiv infection	249 (0.44)
hodgkin’s disease	90 (0.16)
hypercholesterolaemia	88 (0.15)
hyperlipidaemia	70 (0.12)
hypersensitivity	54 (0.09)
hypertension	466 (0.82)
hypothyroidism	98 (0.17)
idiopathic pulmonary fibrosis	123 (0.22)
ill-defined disorder	55 (0.10)
immunodeficiency common variable	186 (0.33)
immunosuppressant drug therapy	85 (0.15)
immunosuppression	100 (0.18)
insomnia	100 (0.18)
juvenile idiopathic arthritis	82 (0.14)
lung neoplasm malignant	91 (0.16)
lupus nephritis	107 (0.19)
malignant melanoma	66 (0.12)
mantle cell lymphoma	106 (0.19)
metastases to bone	50 (0.09)
migraine	99 (0.17)
multiple myeloma	315 (0.55)
multiple sclerosis	4124 (7.24)
myasthenia gravis	121 (0.21)
myelodysplastic syndrome	85 (0.15)
myelofibrosis	285 (0.50)
narcolepsy	116 (0.20)
neuralgia	96 (0.17)
neuropathy peripheral	79 (0.14)
non-hodgkin’s lymphoma	113 (0.20)
non-small cell lung cancer	163 (0.29)
osteoarthritis	73 (0.13)
osteopenia	126 (0.22)
osteoporosis	861 (1.51)
osteoporosis postmenopausal	164 (0.29)
others	8237 (14.45)
ovarian cancer	136 (0.24)
pain	448 (0.79)
parkinson’s disease	72 (0.13)
paroxysmal nocturnal haemoglobinuria	66 (0.12)
peritoneal dialysis	230 (0.40)
plasma cell myeloma	2041 (3.58)
polycythaemia vera	151 (0.26)
post herpetic neuralgia	72 (0.13)
premedication	159 (0.28)
primary immunodeficiency syndrome	81 (0.14)
primary progressive multiple sclerosis	135 (0.24)
product used for unknown indication	7892 (13.85)
prophylaxis	273 (0.48)
prophylaxis against graft *versus* host disease	77 (0.14)
prophylaxis against transplant rejection	106 (0.19)
prostate cancer	200 (0.35)
psoriasis	1598 (2.80)
psoriatic arthropathy	1309 (2.30)
pulmonary arterial hypertension	490 (0.86)
pulmonary hypertension	127 (0.22)
relapsing-remitting multiple sclerosis	796 (1.40)
relapsing multiple sclerosis	266 (0.47)
renal cancer	63 (0.11)
renal cell carcinoma	61 (0.11)
renal transplant	154 (0.27)
rheumatoid arthritis	8802 (15.45)
sarcoidosis	51 (0.09)
secondary progressive multiple sclerosis	70 (0.12)
skin wrinkling	54 (0.09)
stem cell transplant	87 (0.15)
systemic lupus erythematosus	380 (0.67)
type 2 diabetes mellitus	266 (0.47)
unknown	3419 (6.00)
vitamin supplementation	68 (0.12)

### 3.2 Reports and signals detection associated with drug-induced herpes zoster

Based on the frequency of AE reports, Table 3 summarizes the top 30 drugs associated with herpes zoster. Adalimumab (3,577 cases) was the most frequently reported drug, followed by etanercept (3,380 cases), tofacitinib (2,696 cases), infliximab (2,240 cases), lenalidomide (1,639 cases), natalizumab (1,500 cases), ocrelizumab (1,224 cases), rituximab (1,119 cases), fingolimod (1,101 cases), and methotrexate (976 cases). Notably, among these drugs, 24 had herpes zoster risk listed in their package inserts, while the remaining seven did not. These seven drugs were secukinumab, interferon β-1a, human immunoglobulin g, golimumab, alendronate sodium, pregabalin, and ibrutinib.

**TABLE 3:** Top 30 drugs with the highest number of reported herpes zoster.

Drug name	n (%s)	Package insert suggests risk for herpes zoster
adalimumab	3577 (11.95)	Yes
etanercept	3380 (11.29)	Yes
tofacitinib	2696 (9.01)	Yes
infliximab	2240 (7.48)	Yes
lenalidomide	1639 (5.47)	Yes
natalizumab	1500 (5.01)	Yes
ocrelizumab	1224 (4.09)	Yes
rituximab	1119 (3.74)	Yes
fingolimod	1101 (3.68)	Yes
methotrexate	976 (3.26)	Yes
certolizumab pegol	859 (2.87)	Yes
dupilumab	756 (2.53)	Yes
upadacitinib	710 (2.37)	Yes
dimethyl fumarate	675 (2.25)	Yes
Abatacept	663 (2.21)	Yes
tocilizumab	644 (2.15)	Yes
secukinumab	620 (2.07)	No
ruxolitinib	610 (2.04)	Yes
pregabalin	512 (1.71)	No
interferon beta-1a	492 (1.64)	No
human immunoglobulin g	438 (1.46)	No
golimumab	437 (1.46)	No
bortezomib	426 (1.42)	Yes
alendronate sodium	408 (1.36)	No
teriparatide	398 (1.33)	Yes
mycophenolate mofetil	398 (1.33)	Yes
ustekinumab	397 (1.33)	Yes
denosumab	394 (1.32)	Yes
ibrutinib	335 (1.12)	No
mepolizumab	313 (1.05)	Yes

According to the ROR criteria, Table 4 lists the top 30 drugs with the strongest signal strength. Anifrolumab (n = 45; ROR 20.97, PRR 19.87, χ2 807.81) had the highest ROR for herpes zoster, followed by rozanolixizumab (n = 7; ROR 16.05, PRR 15.4, χ2 94.53), ocrelizumab (n = 1,224; ROR 9.64, PRR 9.42, χ2 9010.61), alemtuzumab (n = 286; ROR 9.34, PRR 9.13, χ2 2063.41), tofacitinib (n = 2,696; ROR 8.27, PRR 8.11, χ2 15942.94), pamidronate disodium (n = 86; ROR 8.2, PRR 8.04, χ2 530.5), baricitinib (n = 122; ROR 7.88, PRR 7.74, χ2 715.77), tozinameran (n = 16; ROR 7.56, PRR 7.43, χ2 89.18), elapegademase (n = 4; ROR 7.15, PRR 7.03, χ2 20.75), and satralizumab (n = 13; ROR 6.18, PRR 6.09, χ2 55.42). Among the top 30 drugs, 18 had herpes zoster risk identified in their package inserts, while the remaining 12 did not.

**TABLE 4:** Top 30 drugs for signal strength.

Drug name	Case reports	ROR (95% CI)	PRR (95% CI)	χ2	Package insert suggests risk for herpes zoster
anifrolumab	45	20.97 (15.53, 28.33)	19.87 (15.1, 26.14)	807.81	Yes
rozanolixizumab	7	16.05 (7.53, 34.23)	15.4 (7.46, 31.8)	94.53	No
ocrelizumab	1224	9.64 (9.1, 10.21)	9.42 (8.88, 9.99)	9010.61	Yes
alemtuzumab	286	9.34 (8.3, 10.5)	9.13 (8.12, 10.27)	2063.41	Yes
tofacitinib	2696	8.27 (7.95, 8.6)	8.11 (7.8, 8.43)	15942.94	Yes
pamidronate disodium	86	8.2 (6.62, 10.15)	8.04 (6.48, 9.97)	530.5	Yes
baricitinib	122	7.88 (6.59, 9.43)	7.74 (6.49, 9.23)	715.77	Yes
tozinameran	16	7.56 (4.61, 12.41)	7.43 (4.55, 12.13)	89.18	No
elapegademase	4	7.15 (2.66, 19.25)	7.03 (2.64, 18.73)	20.75	No
satralizumab	13	6.18 (3.57, 10.69)	6.09 (3.52, 10.54)	55.42	No
efgartigimod alfa-fcab	47	5.99 (4.49, 8)	5.91 (4.49, 7.78)	192.2	No
upadacitinib	710	5.92 (5.49, 6.38)	5.84 (5.4, 6.32)	2816.11	Yes
pralatrexate	9	5.82 (3.01, 11.25)	5.74 (3.01, 10.96)	35.35	No
abrocitinib	26	5.16 (3.5, 7.6)	5.1 (3.51, 7.4)	85.97	Yes
ibritumomab tiuxetan	13	5.13 (2.97, 8.86)	5.07 (2.93, 8.78)	42.56	Yes
bortezomib	426	5.03 (4.57, 5.54)	4.98 (4.52, 5.49)	1345.52	Yes
fingolimod	1101	4.96 (4.67, 5.27)	4.91 (4.63, 5.21)	3360.5	Yes
infliximab	2240	4.9 (4.7, 5.11)	4.85 (4.66, 5.04)	6558.82	Yes
arsenic trioxide	16	4.87 (2.97, 7.98)	4.82 (2.95, 7.87)	48.54	Yes
cladribine	69	4.72 (3.72, 5.98)	4.67 (3.69, 5.91)	199.14	Yes
ciclesonide	20	4.54 (2.92, 7.06)	4.5 (2.92, 6.93)	54.54	No
mepolizumab	313	4.53 (4.05, 5.07)	4.49 (3.99, 5.05)	845.92	Yes
efalizumab	32	4.46 (3.14, 6.32)	4.41 (3.1, 6.28)	84.69	No
ambrosia artemisiifolia pollen	3	4.36 (1.4, 13.62)	4.32 (1.41, 13.2)	7.68	No
alendronate sodium	408	4.35 (3.95, 4.8)	4.31 (3.91, 4.75)	1032.71	No
rituximab	1119	4.35 (4.1, 4.62)	4.31 (4.06, 4.57)	2787.82	Yes
pentoxifylline	6	4.34 (1.94, 9.72)	4.3 (1.93, 9.6)	15.26	No
glofitamab	6	4.33 (1.93, 9.68)	4.29 (1.92, 9.58)	15.16	Yes
anakinra	93	4.29 (3.49, 5.26)	4.25 (3.49, 5.17)	231.29	No
tocilizumab	644	4.2 (3.88, 4.54)	4.16 (3.85, 4.5)	1529.86	Yes

## 4 Discussion

This study comprehensively evaluated real-world adverse event reports related to drug-induced herpes zoster using the FAERS database. The clinical characteristics of these events were detailed, and the drugs most strongly associated with herpes zoster were identified. Notably, many of these drugs lacked warnings for herpes zoster on their labels, leading to insufficient awareness of their risks. The occurrence of herpes zoster can severely impact patients’ health, highlighting the need for heightened awareness among healthcare professionals.

During the study, significant age and gender differences were observed in all reported drug-related herpes zoster cases to the FDA. The reporting frequency was notably higher in females than in males, and 57.05% of herpes zoster cases occurred in individuals over 40 years old. This may be because females are more susceptible to chronic diseases requiring immunosuppressants or other high-risk medications (Mok et al., 2020), and being female is an independent risk factor for herpes zoster, particularly evident in middle age (Opstelten et al., 2006). Interestingly, most adverse reaction reports (43.22%) were submitted by consumers rather than medical professionals, suggesting that patients may be more inclined to report adverse reactions directly or that healthcare professionals might underreport. Furthermore, most reports originated from the United States (50.67%), indicating potential regional or cultural differences in reporting practices that warrant further investigation. Apart from unspecified adverse events, the most common adverse events were death and hospitalization, underscoring the severe impact of drug-related herpes zoster. The primary indications for drug-related herpes zoster included multiple sclerosis and rheumatoid arthritis, consistent with the routine use of high-risk drugs (such as Ocrelizumab, Alemtuzumab, Satralizumab, Tofacitinib, and Baricitinib) in these patient populations (McGinley et al., 2021; Miyazaki et al., 2021).

Among the 30 most frequently reported drugs associated with herpes zoster, seven were not listed on package insert labels. Notably, secukinumab, an IL-17A inhibitor, is primarily used to treat psoriasis, psoriatic arthritis, and ankylosing spondylitis. A review of previous literature revealed that psoriasis patients receiving secukinumab treatment had a higher association with herpes simplex but did not exhibit an increased risk of HZ infection (Davidson et al., 2022). Most studies reported few or no cases of HZ in patients receiving IL-17 therapy (Higashi et al., 2024). Secukinumab may weaken the body’s immune response to viral infections by inhibiting the IL-17 pathway, thereby triggering herpes zoster, a point not mentioned in the drug’s label. Pregabalin, a γ-aminobutyric acid analog, is commonly used to treat neuropathic pain, epilepsy, and generalized anxiety disorder. Although generally well-tolerated, its main adverse reactions include dizziness, somnolence, and weight gain (Fuzier et al., 2013), with no reports of inducing herpes zoster. However, pregabalin’s analgesic effect may mask the early symptoms of herpes zoster, delaying diagnosis and treatment (Yamamoto et al., 2018). Interferon β-1a is one of the first approved disease-modifying therapies for relapsing multiple sclerosis and has also demonstrated broad antiviral properties (Cohen et al., 2012; Salvetti et al., 2024). In clinical trials, the most common adverse events of interferon β-1a were mild to moderate injection site reactions, flu-like symptoms, fever, and headache (Kolb-Mäurer et al., 2019). Although interferons are generally considered to modulate antiviral responses and have been used to treat viral infections (Bellucci et al., 2023), their potential risk of inducing herpes zoster has not been fully recognized. Human immunoglobulin G is primarily used to treat primary and secondary immunodeficiencies, neuromuscular diseases, and Kawasaki disease (Guo et al., 2018). Although it is generally well-tolerated, some patients may have an increased risk of infection during treatment (Guo et al., 2018), but herpes zoster is rarely emphasized. Golimumab, an anti-TNF-α fully human monoclonal antibody, has been FDA-approved for treating various autoimmune diseases, including rheumatoid arthritis and ankylosing spondylitis. Golimumab is generally well-tolerated, but its associated adverse reactions include an increased risk of infection, injection site reactions, and rare serious allergic reactions (Yang and Kavanaugh, 2014). Upper respiratory tract infections are the most common type of infection triggered by golimumab, while herpes zoster, as a potential adverse reaction, may not have received sufficient attention due to its lower incidence (Kim et al., 2021). Alendronate sodium, a bisphosphonate drug, is used to treat osteoporosis and other metabolic bone diseases. Its adverse reactions typically include gastrointestinal discomfort, bone and joint pain (Biswas et al., 2003). Although osteoporosis patients may face an increased risk of infection due to decreased immune function, the mechanism by which alendronate sodium triggers herpes zoster remains unclear, and this signal requires further validation. Ibrutinib, a Bruton’s tyrosine kinase (BTK) inhibitor, is widely used to treat hematologic malignancies such as mantle cell lymphoma and chronic lymphocytic leukemia. The known adverse reactions of ibrutinib include the risk of bleeding, infection, arrhythmias, and hypertension (Byrd et al., 2014). Previous studies have reported herpes zoster infections in chronic lymphocytic leukemia patients receiving ibrutinib treatment (Okay et al., 2019; Coutre et al., 2019), but the specific mechanism warrants further investigation.

Based on the disproportionality analysis, we identified the top 30 drugs associated with the risk of herpes zoster and compared their signal strength with label information. Among these 30 drugs, 18 had herpes zoster risk mentioned in their drug labels, whether classified as common or rare adverse events (AEs), with most being immunosuppressants. These drugs exert their therapeutic effects by suppressing the body’s immune function, which may also weaken the body’s ability to defend against viral infections, thereby increasing the risk of herpes zoster (Kawai et al., 2014). For example, SLE and multiple sclerosis treatments such as anifrolumab, ocrelizumab, alemtuzumab, and fingolimod, as well as rheumatoid arthritis treatments like infliximab and tocilizumab, all include warnings about herpes zoster risk in their labels. Additionally, some novel selective immunosuppressants like baricitinib, upadacitinib, abrocitinib, and tofacitinib, despite having higher efficacy and safety, still inevitably carry a certain risk of opportunistic infections (Xu et al., 2023).

Radioimmunoconjugate drugs such as ibritumomab, anti-tumor drugs like bortezomib, arsenic trioxide, and cladribine also mention herpes zoster risk in their labels. Most of these drugs have myelosuppressive effects, leading to neutropenia and decreased immunity, which increases the chance of viral infections (Cook et al., 2019; Schiller et al., 2006). Apart from immunosuppressants and anti-tumor drugs, some medications for specific diseases, such as the bisphosphonate osteoporosis drug pamidronate disodium and the hypereosinophilic syndrome treatment mepolizumab, also have herpes zoster risk warnings in their labels. Overall, any drug with immunosuppressive effects or that can cause immune dysfunction may potentially increase the risk of herpes zoster. Healthcare professionals should carefully weigh the treatment benefits and infection risks when selecting treatment plans for patients and provide necessary preventive measures for high-risk populations.

Among the remaining 12 drugs with strong signals but no mention of herpes zoster risk in their labels, most also have immunosuppressive or immunomodulatory effects, such as rozanolixizumab, satralizumab, efgartigimod, efalizumab, and anakinra. Although their labels do not explicitly state infection risks, based on their mechanisms of action and the usage experience of other similar drugs, clinicians and patients should remain vigilant. Furthermore, some drugs for rare diseases or malignancies like elapegademase and pralatrexate, as well as corticosteroids like ciclesonide for specific diseases, although lacking clear evidence of inducing herpes zoster risk, should also raise risk awareness among patients considering the increased possibility of herpes zoster under immunocompromised and stressful conditions.

This study also found associations between some special drugs and the risk of herpes zoster. For example, Ambrosia artemisiifolia pollen extract, used for pollen allergy desensitization treatment, may affect the body’s immune balance with long-term use, although the mechanism remains unclear. Pentoxifylline, used to treat peripheral vascular diseases, may also influence susceptibility to viral infections through its immunomodulatory effects. Additionally, the COVID-19 mRNA vaccine tozinameran, although inconclusive regarding infection risk, should be included in the scope of attention considering that COVID-19 infection itself can trigger herpes zoster (van Dam et al., 2021), and the body may be in a stressed state with temporarily affected immune function after vaccination.

Potential Underreporting and Lack of Attention to Herpes Zoster Risk Many of the drugs identified in this study have been on the market for several years, yet their potential association with herpes zoster has not received sufficient attention. According to signal detection criteria, three or more reports of herpes zoster can be considered a signal, but this may lead to false-positive results. Among the 12 drugs that may generate new signals in our analysis, only tozinameran (van Dam et al., 2021), efalizumab (Tang et al., 2021), and anakinra (Navarro et al., 2021) have related reports, while no obvious herpes zoster cases have been found for the other drugs. Therefore, whether the package inserts of these drugs need to include warnings about herpes zoster risk requires further discussion. As a key legal document, the drug package insert is not only an important channel for patients to understand adverse reactions, but its information gaps may also pose serious threats to patient health.

Regarding signal detection methods, frequentist approaches often have high sensitivity but are also prone to false-positive signals (Zonta et al., 2014). Nevertheless, this method can still be used to validate hypotheses initially formed through clinical observation and literature analysis. For example, the package insert of tramadol does not mention the risk of hypoglycemia, but both the literature and the study by Juba et al. based on the FAERS database have confirmed this association (Li et al., 2024).

This study also presents some limitations that should be acknowledged. First, although disproportionality analysis helps to determine a statistical method for identifying associations between targeted drugs and adverse events, it does not establish a clear causal relationship between the targeted drugs and adverse reactions. Second, although we conducted subgroup analyses based on age, gender, country, and disease in this study, the data is derived from the FAERS database, which relies on voluntary and spontaneous reporting. This may be influenced by recent research or media reports, potentially introducing bias and distorting the true incidence of herpes zoster (Maciá-Martínez et al., 2016). Furthermore, the lack of clear classification of cases (such as specific subtypes of herpes zoster) limits the depth of the analysis. Future research can combine electronic health records or other databases and validate signals through prospective studies to improve the reliability of the results. Despite these limitations, this study provides an important basis for identifying high-risk drugs and developing pharmacovigilance strategies, which can help optimize clinical decision-making and patient safety.

## 5 Conclusion

This study comprehensively evaluated herpes zoster reports and associated drugs using the FAERS database, identifying several high-risk drugs, some of which are not currently listed as having a risk of herpes zoster in their package inserts. These findings emphasize the need for heightened clinical vigilance, particularly when prescribing immunosuppressants and immunomodulators. Specifically, drugs like anifrolumab, rozanolixizumab, and ocrelizumab showed strong statistical associations with herpes zoster, warranting further investigation. Clinicians should consider the potential for increased HZ risk when using these and other identified medications, and prophylactic antiviral therapy or vaccination may be considered for high-risk patients. While this study provides valuable insights from real-world data, it is important to acknowledge that disproportionality analysis does not establish causality. Prospective studies are needed to confirm these associations, quantify absolute risk, and elucidate the underlying mechanisms. This study underscores the critical role of pharmacovigilance and post-marketing surveillance in identifying potential drug safety signals and enhancing patient safety. By leveraging real-world data, we can improve our understanding of drug-induced adverse events and optimize clinical decision-making.

## Data Availability

The original contributions presented in the study are included in the article/supplementary material, further inquiries can be directed to the corresponding author.
